# Supplementary material for: Super-shear ruptures steered by pre-stress heterogeneities during the 2023 Kahramanmaraş earthquake doublet
Source: Nat Commun. 2024 Aug 14;15:7004. doi: 10.1038/s41467-024-51446-y (PMC11325041; doi:10.1038/s41467-024-51446-y)
Supplement: Supplementary file 1 — Supplementary Information [file 41467_2024_51446_MOESM1_ESM.pdf]

Supplementary Information for

Super-shear ruptures steered by pre-stress heterogeneities during the 2023 Kahramanmaraş earthquake doublet

Kejie Chen[1*], Guoguang Wei[1], Christopher Milliner[2], Luca Dal Zilio[3,4], Cunren Liang[5], Jean-Philippe Avouac[2]

1 Department of Earth and Space Sciences, Southern University of Science and Technology, Shenzhen, China

2 Division of Geological and Planetary Sciences, California Institute of Technology, Pasadena, USA

3 Earth Observatory of Singapore, Nanyang Technological University, Singapore, Singapore

4 Asian School of the Environment, Nanyang Technological University, Singapore, Singapore

5 School of Earth and Space Sciences, Peking University, Beijing, China

*Corresponding author: Kejie Chen (chenkj@sustech.edu.cn)

Contents of this file

Supplementary Notes 1 to 3
Supplementary Tables 1 to 5
Supplementary Figures 1 to 26
Supplementary References

Supplementary Note 1 and 2 describe how the 2D and 3D co-seismic deformation fields are calculated from radar and optical images, Supplementary Note 3 details how the pre-stress tensor is inverted. Supplementary Table 1 summarizes the satellte image information. Supplementary Table 2 lists prior distributions of fault geometry parameters, Supplementary Table 3 is a 1-D velocity structure model and Supplementary Table 4 summarizes the estimated dip angles for all segments. Supplementary Table 5 provides the mean fault strike from the slip model and the SHmax. Supplementary Figures 1-6 present co-seismic 3D deformation fileds and interferograms, while Supplementary Figures 7-11 show data aopted and results realted to Bayesian inversion. Supplementary Figures 12 -19 display data fits, sub-fault source time functions and Jackknife tests for the preferred kinematic models of the M7.8 and M7.5 events. Supplementary Figure 20 presents the data fits if only westward supershear is assumed for the M7.5 event. Supplementary Figures 21-24 demonstrate stress model and Supplementary Figure 25 illustrates NPF-EAF region with background information. Supplementary Figure 26 is the enlarged pre-stress tensors and fault-slip data for different zones.

Supplementray Note 1. 2D horizontal deformation from subpixel correlation

To determine the 2D horizontal deformation field, we used a subpixel correlation technique on optical images acquired before and after the M7.8 and M7.5 earthquakes. These orthorectified images were obtained from the European Space Agency's Sentinel-2 satellite sensor that acquires images at 10 m pixel resolution. Specifically, the deformation maps were estimated from the images using the COSI-Corr software which uses a phase correlation scheme to estimate the sub-pixel shift of a subset of pixels under the assumption that a translation in the spatial domain is equivalent to a shift in phase [1,2]. Here we used a correlation window with a dimension of 64×64 pixels and a step size of 16 pixels. This produces a 2D deformation map of the horizontal surface motion with a uniform pixel resolution of 160 m.

We used right-looking C-band Sentinel-1 SAR images acquired before and after the M7.8 and M7.5 events to estimate radar amplitude pixel offsets. In total we used data from three tracks, two ascending (Track 14 and 116) and one descending (Track 21) that were acquired in TOPS mode. Together these tracks cover the entire rupture constraining surface motion in the range and azimuthal (i.e., in-flight) directions. To estimate the pixels offsets we used the InSAR Scientific Computing Environment (ISCE-2) developed at the Jet Propulsion Lab to first co-register the reference and secondary single-look complex images (SLCs) [3]. For cross-correlation we used a window of 64×64 pixels, a search distance of 20 pixels and a skip size of 32 pixels, using the denseOffsets.py routine in ISCE. Following correlation, the pixel offsets are geocoded from radar to geographic co-ordinates. The variability for each of the radar offset results is listed in Supplementary Table 1, which is estimated from the variability in surface motion of a far-field stable region.

Supplementray Note 2. 3D surface deformation from inversion of radar and optical pixel offsets

To align the various datasets into the same co-ordinate system, the offsets are taken relative to a far-field stable point that is common to all tracks (37.0209° E, 37.0172° N) instead of using GNSS as there is insufficient station coverage located across each track. This gives a surface deformation result that is in a reference frame relative to the stable region.

The 3D surface deformation (m) is then estimated at each pixel from inverting the satellite pixel offsets (d)

with at least three independent look directions using a weighted least squares (WLS) approach, $m = (G'WG)^{-1}G'Wd$. The diagonal weighting matrix (W) contains values that are the inverse of the variances of each dataset (with 1σ uncertainty up to 0.2 m and 0.5 m for Sentinel-1 range and azimuths respectively, while we find uncertainty of 1σ = 0.25 m and 0.31 cm for Sentinel-2 east-west and north-south components respectively, see Supplementary Table 1 for details). Measurements of the surface fault slip show very strong statistical agreement with measurements from other studies using similar pixel tracking geodetic imaging data (Supplementary Figure 3).

Supplementtray Note 3. Prestress tensor inversion

The 3D deviatoric stress tensor is estimated by inverting the focal mechanisms from *Güvercin et al.* [4] under the Wallace-Bott assumption that slip is parallel to the shear stress [5]. The stress inversion gives the orientation and shape of the 3D deviatoric stress tensor but not its magnitude. The principal deviatoric stresses $\sigma_1, \sigma_2, \sigma_3$ are ordered from most to least compressive. To resolve spatial variations of stress along the rupture we distinguish three zones along the M7.8 rupture (Supplementary Figure 21), making sure that each contains sufficient diversity of fault orientation to resolve the stress tensor according to the criteria given by *Hardebeck and Hauksson* [6]. Where for all zones the angle of the slip vectors have an RMS > 30° from the average (see Supplementary Figure 22 which shows the distributions of each zone). To determine the nodal plane for our inversion for the stress tensors, we follow the approach of *Vavryčuk* [7] by using an iterative approach to pick nodal planes that are optimally aligned to the stress field that have the highest fault instability value.

To invert the unit slip vectors for stress we use a L2 least squares inversion [8]. To minimize overfitting of the data and to constrain stress to be spatially smooth along the rupture we use a damping constraint to the inversion that penalizes large changes of the stress orientation between neighboring zones (i.e., the gradients of the model vector between cells) [9]. The uncertainties (see Supplementary Figure 22) of the stress model are then estimated from bootstrapping via random replacement of the original unit slip vectors (Supplementary Figure 23), and the angular misfits are shown in Supplementary Figure 24.

Supplementary Table 1 Information of Sentinel-1 radar and Sentinel-2 optical images

Satellite	Sensor type	orbit direction	Track (T)/Relative orbit (RO) ID	look direction	1σ far-field (meters)
Sentinel-1	radar	descending	T21	range	0.1
Sentinel-1	radar	descending	T21	azimuth	0.4
Sentinel-1	radar	ascending	T14	range	0.1
Sentinel-1	radar	ascending	T14	azimuth	0.43
Sentinel-1	radar	ascending	T116	range	0.18
Sentinel-1	radar	ascending	T116	azimuth	0.53
Sentinel-2	optical	N/A	RO121	east-west	0.25
Sentinel-2	optical	N/A	RO 121	north-south	0.31

Supplementary 2 Prior distributions of fault geometry parameters

Model parameter	Prior PDF
Dip angle	$U(30°, 90°)$
Width	$U(0.1\ km, 30\ km)$
Strike-slip	$U(-10\ m, 10\ m)$
Dip-slip	$U(-4\ m, 4\ m)$

Supplementary Table 3 The 1D velocity model used in this study

Depth (km)	Vp(km/s)	Vs(km/s)
0	3.88	2.04
1	4.52	2.43
2	5.62	3.03
4	5.75	3.31
6	5.85	3.38
8	5.96	3.43
10	6.00	3.44
12	6.05	3.46
16	6.32	3.62
20	6.40	3.67
25	6.83	3.92
30	6.89	3.94
37	7.80	4.40
45	8.22	4.56
60	8.30	4.61

Supplementary Table 4 Dip angles for all fault segments

Segment number	Segment start position (Longitude, Latitude)	Segment end position (Longitude, Latitude)	Segment dip angle
A1	36.16°E, 36.14°N	36.44°E, 36.68°	80±5°
A2	36.44°E, 36.68°N	36.64°E, 37.10°	85±3°
A3	36.64°E, 37.10°N	36.90°E, 37.40°	84 ±3°
A4	36.90°E, 37.40°N	37.21°E, 37.53°	89±2°
A5	37.21°E, 37.53°N	37.62°E, 37.78°	86±1°
A6	37.62°E, 37.78°N	38.17°E, 37.98°	89±2°
A7	38.17°E, 37.98°N	38.56°E, 38.10°N	89±4°
A8	37.21°E, 37.53°N	37.06°E, 37.14°N	73°
A9	37.62°E, 37.78°N	37.94°E, 37.83°N	90°
B1	36.41°E, 37.98°N	36.73°E, 38.06°N	52±4°
B2	36.73°E, 38.06°N	37.03°E, 38.05°N	69±2°
B3	37.03°E, 38.05°N	37.35°E, 38.00°N	64±1°
B4	37.35°E, 38.00°N	37.63°E, 37.98°N	72±2°
B5	37.63°E, 37.98°N	38.10°E, 38.21°N	54±4°
B6	36.73°E, 38.06°N	36.71°E, 37.86°N	54±3°

Note: the dip angle estimates and uncertainties are given by the median and standard deviation of the sample distribution, respectively

Supplementary Table 5 Mean fault strike from the slip model and the *SHmax*

	Southern Zone	Central Zone	Northern Zone
SHmax (°)	355.7	2.8	20.6
Mean fault strike (°)	22.6	47.9	67.2

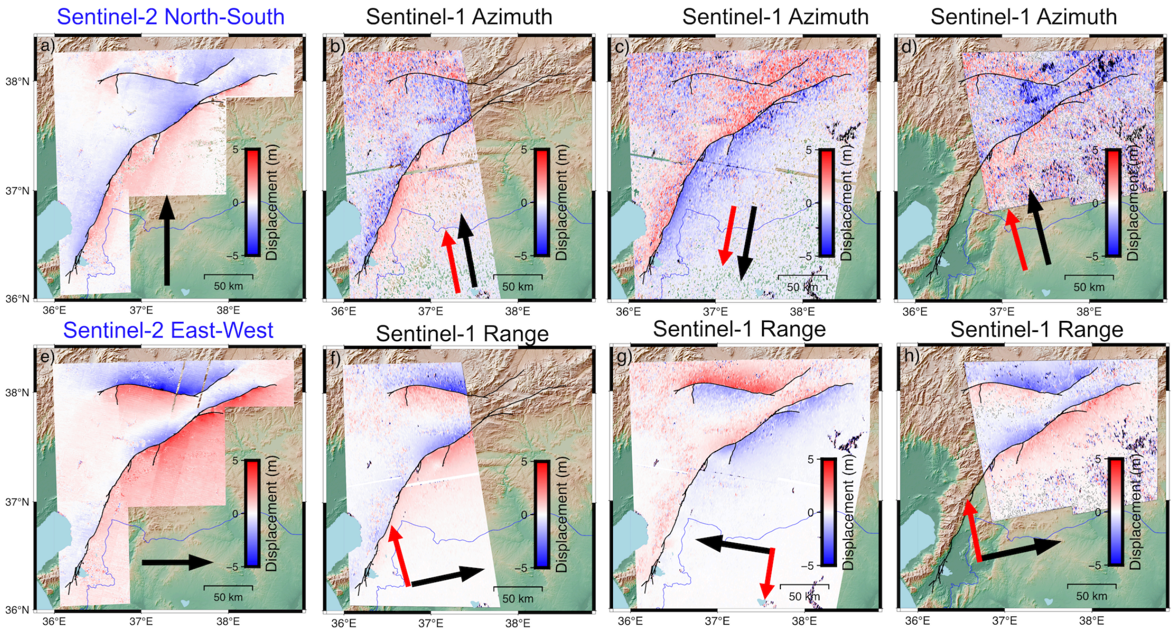

Supplementary Figure 1. The optical (Sentinel-2) and radar (Sentinel-1) pixel offset data used to invert for the 3D surface deformation. The black arrows show the direction of motion measured from the pixel offset, and red shows the orbit direction (not shown for optical).

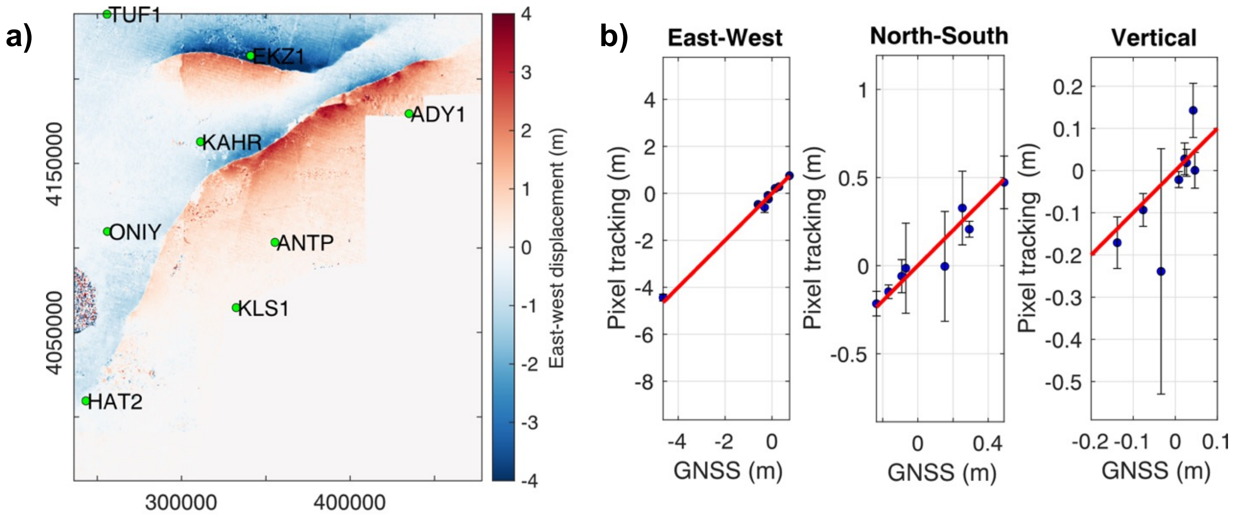

Supplementary Figure 2. Co-seismic deformation and surface displacement comparisons. a) Co-seismic east-west deformation inverted from the Sentinel-1 and Sentinel-2 satellite image offsets. b) Comparisons between the coseismic surface displacement from the 3D deformation geodetic imaging data (shown in Fig. 2a) and the GNSS records from University of Nevada Reno [10].

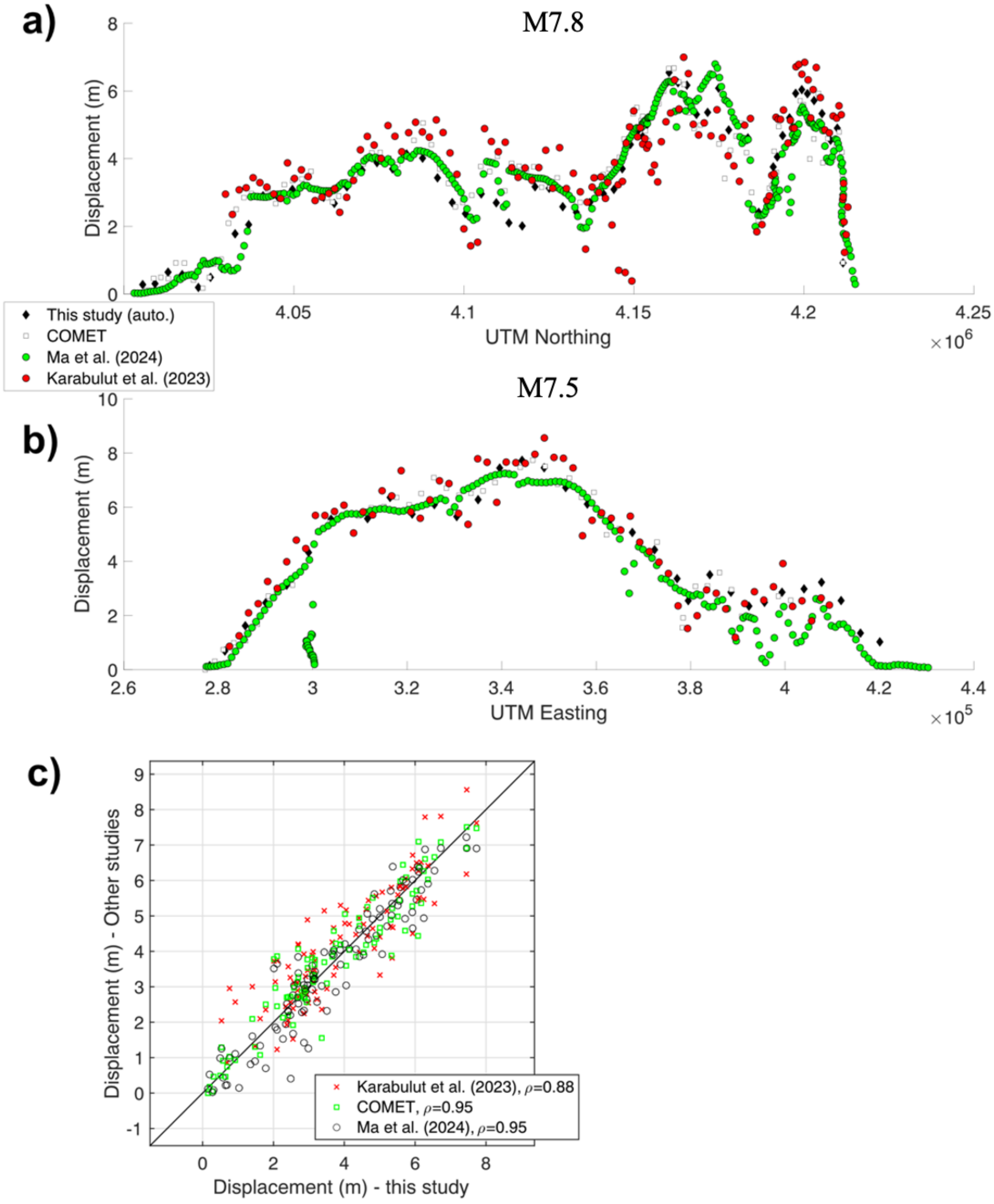

Supplementary Figure 3. Comparisons between fault slip measured from 3D surface displacement maps and other studies. Here we compare fault slip to that of *Karabulut et al.* [11], *Ma et al.* [12] [12]and a dataset from COMET [13] for the M7.8 mainshock (a) and the M7.5 aftershock (b). c) shows a correlation plot between our measurements of surface fault slip and that of previous studies with correlation coefficients show in the lower right.

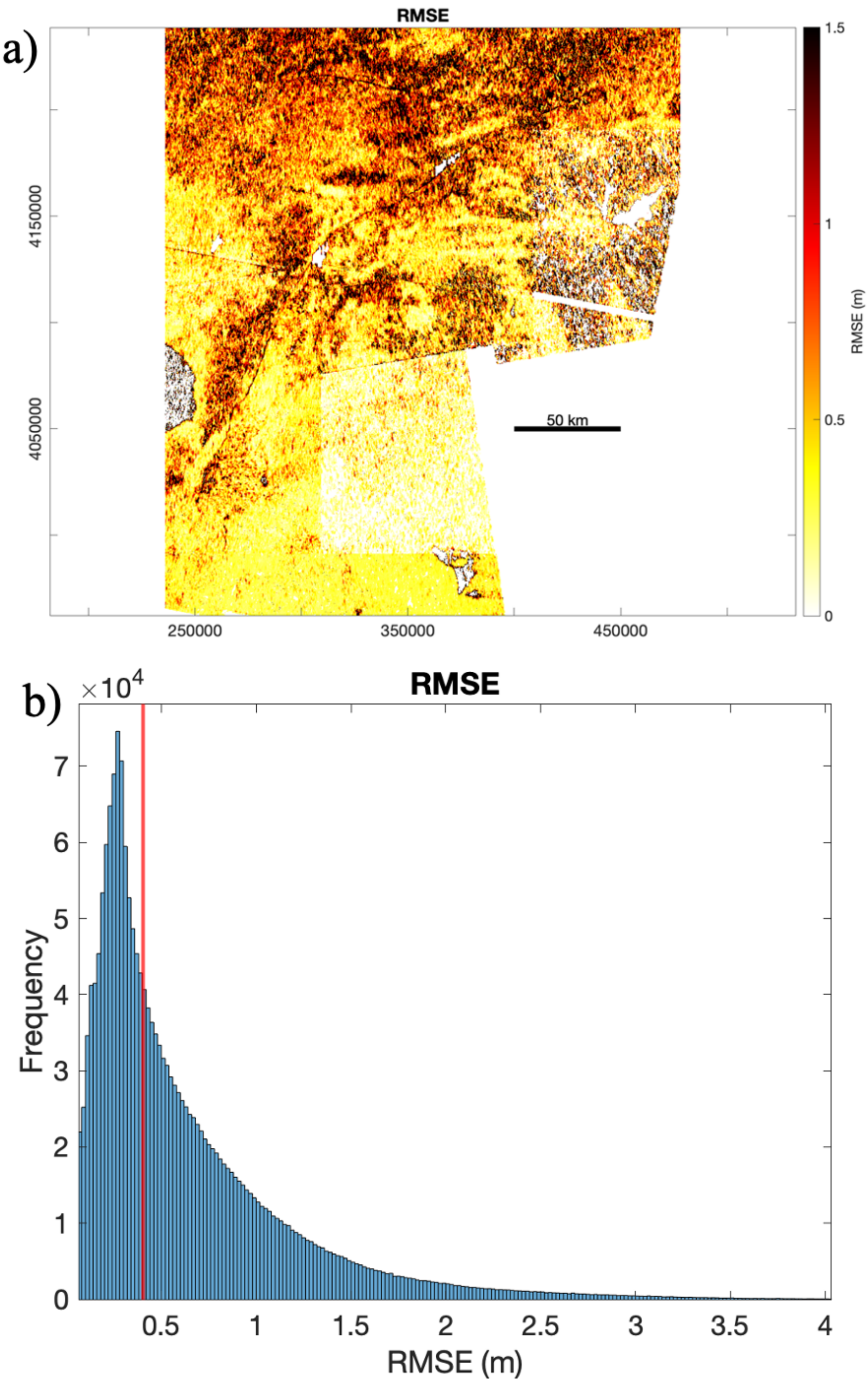

Supplementary Figure 4. Root Mean Square Error (RMSE) of the pixel offsets. a) shows RMSE at each pixel and b) summarizes the frequency distribution with 0.40 m as median for the entire region.

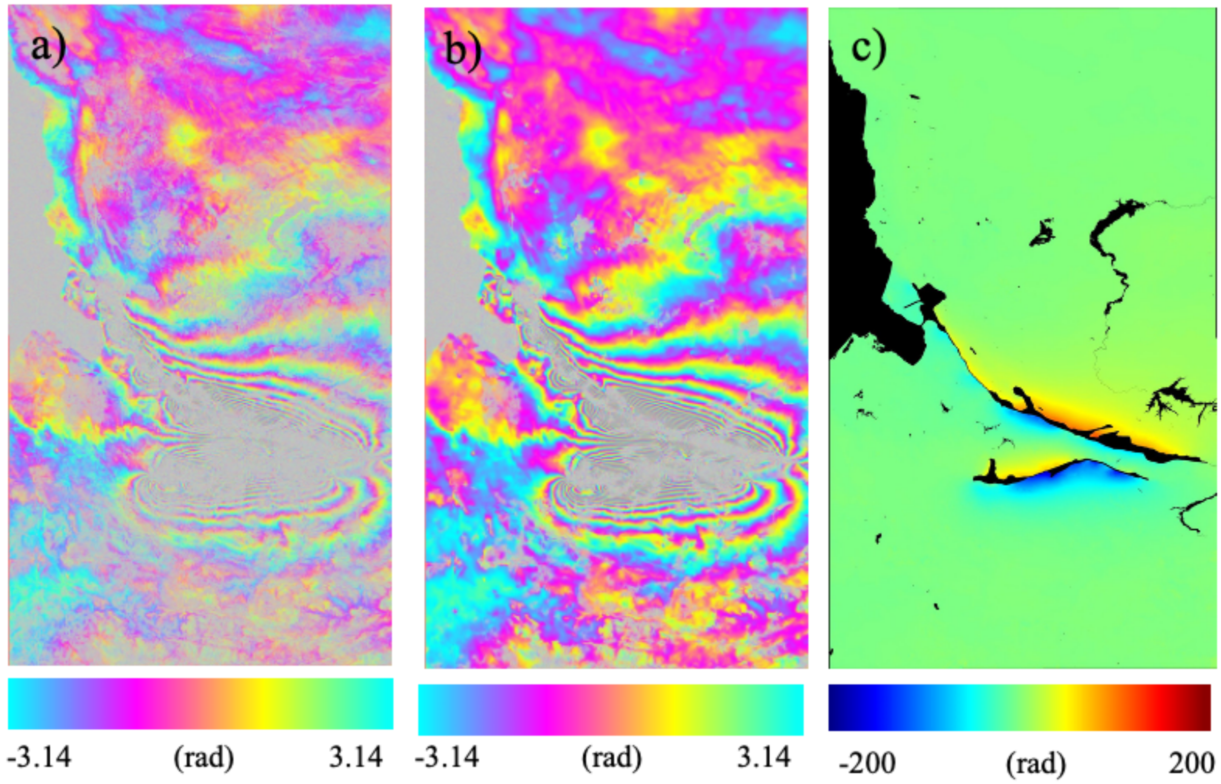

Supplementary Figure 5. ALOS-2 ascending interferograms. ALOS-2 ascending original a) and filtered b) interferograms, c) shows the unwrapped interferograms without any phase wrapping or geoencoding.

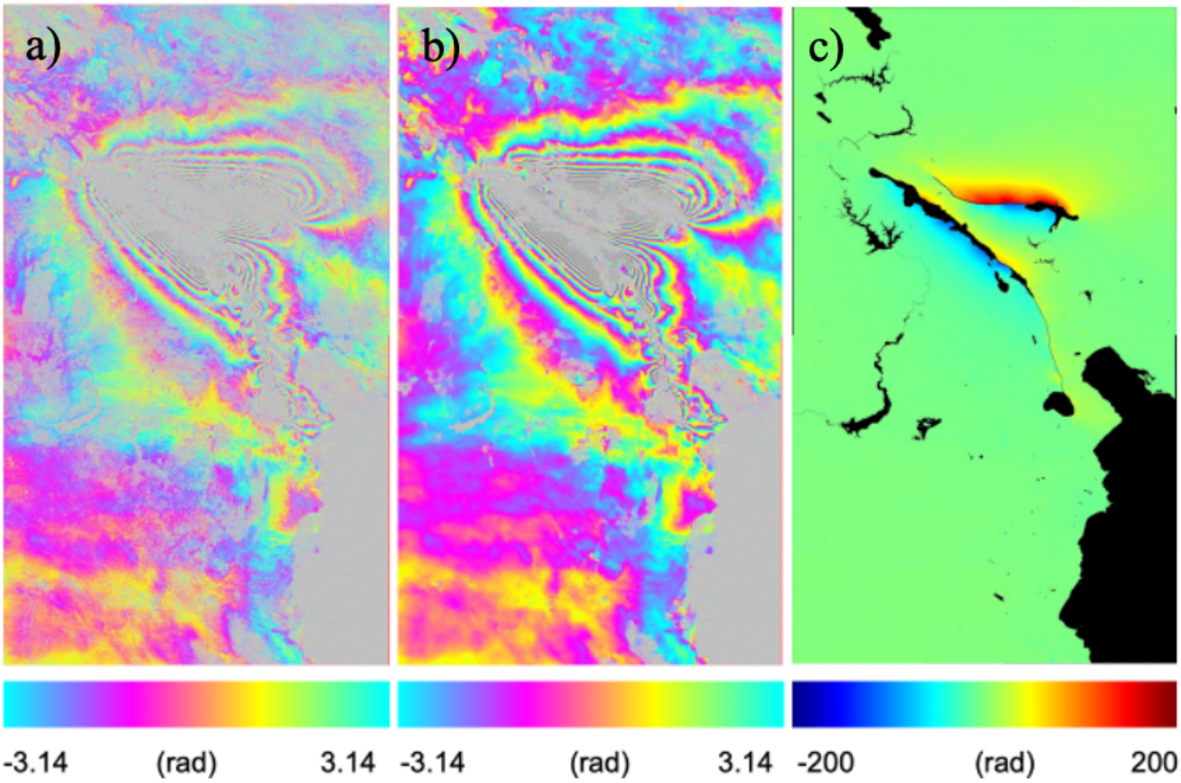

Supplementary Figure 6. ALOS-2 descending interferograms. ALOS-2 descending original a) and filtered b) interferograms, c) shows the unwrapped interferograms without any phase wrapping or geoencoding.

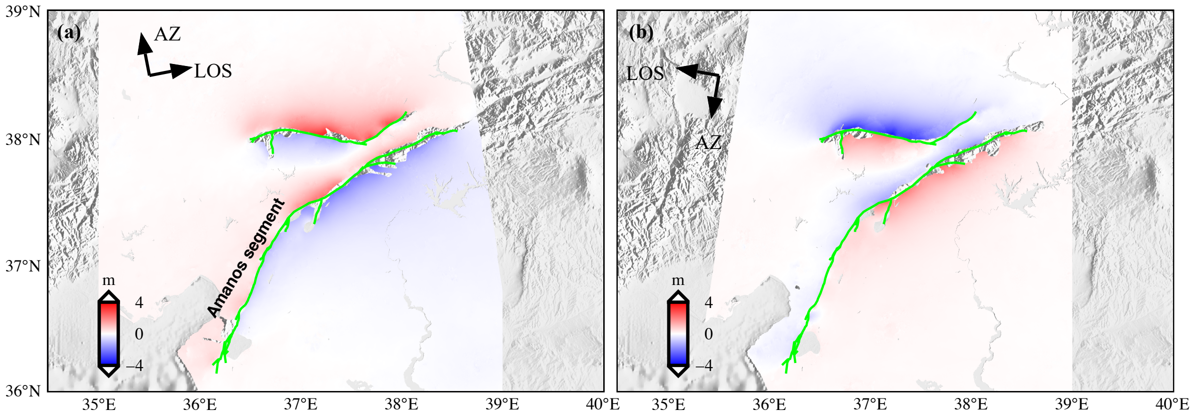

Supplementary Figure 7. ALOS-2 LOS displacement maps. ALOS-2 LOS displacement (positive toward satellite) maps from the ascending track (a) and the descending track (b), respectively. The green lines show the surface rupture traces inferred by the SAR 3D deformation.

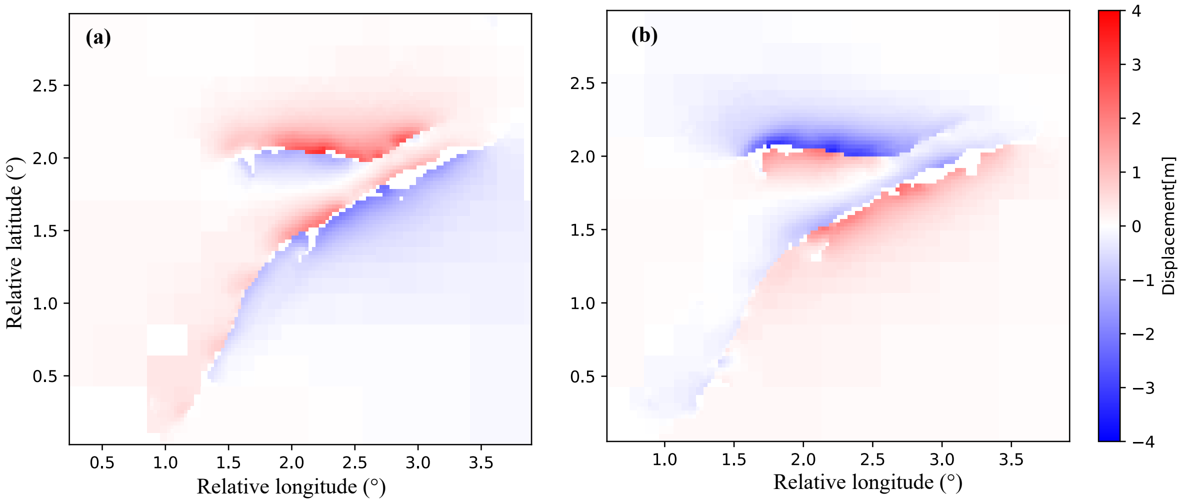

Supplementary Figure 8. Quadtrees for down-sampling ALOS-2 LOS displacement maps. Quadtrees applied for down-sampling ALOS-2 LOS displacement maps for the ascending track (a) and the descending track (b), respectively. The longitude and latitude have been shifted with respect to the reference point (35° E, 36°N).

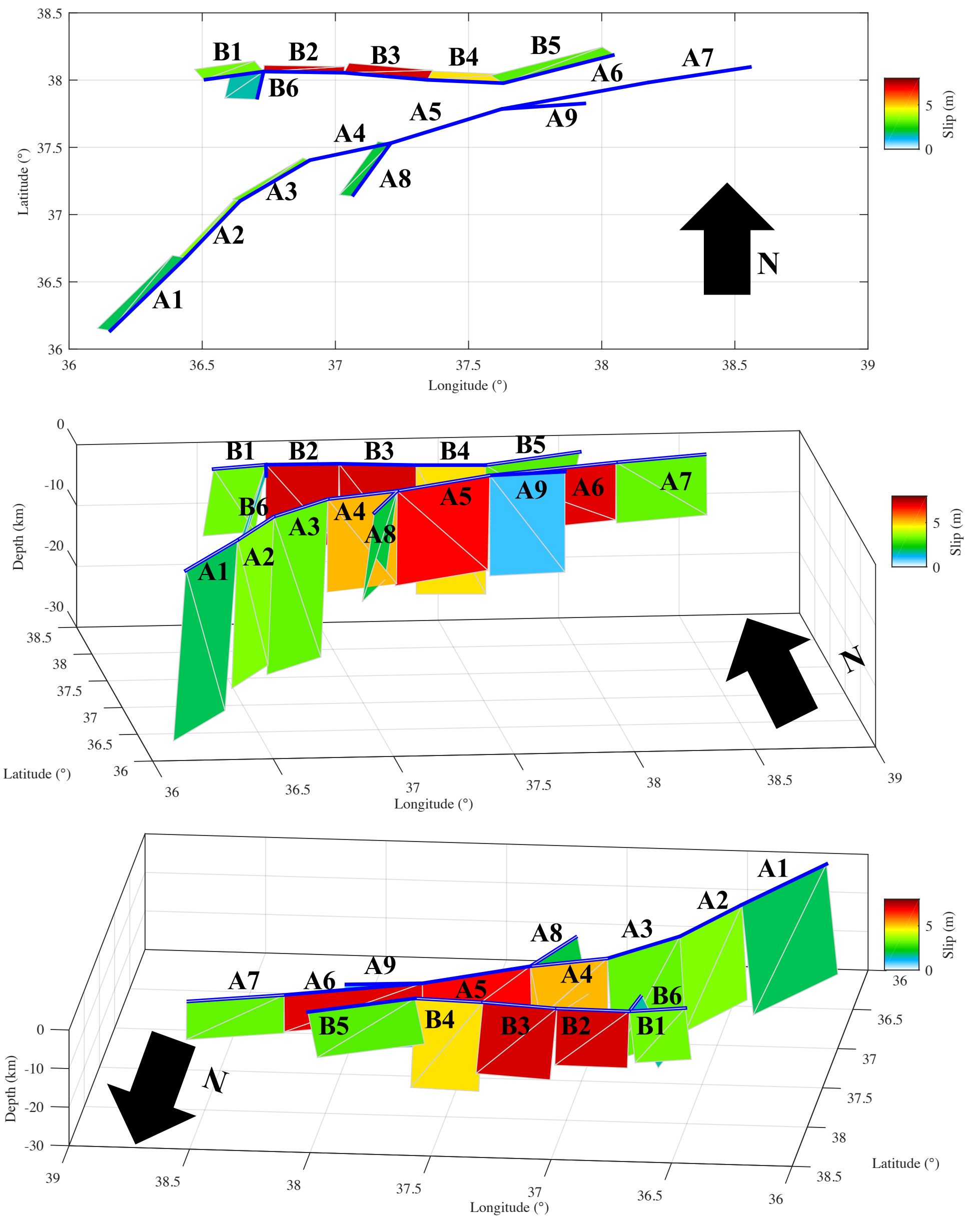

Supplementary Figure 9. 3-D view of fault geometry and uniform slip from different perspectives. 3-D view of fault geometry and uniform slip on each segment, estimated by the medians of their posterior samples from the Bayesian inversion. N denotes the north direction.

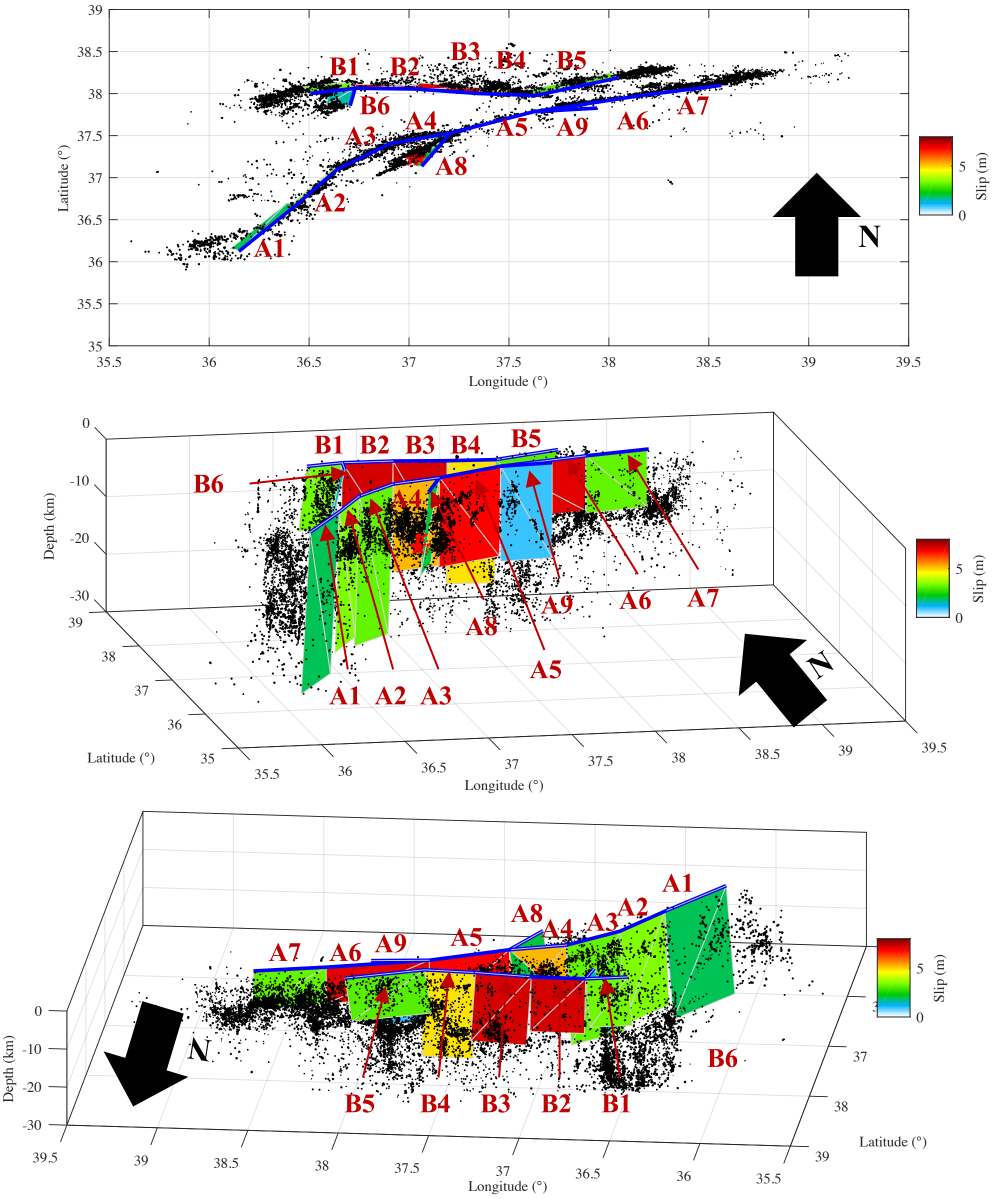

Supplementary Figure 10. **3-D fault geometry, slip distribution, and aftershocks from different perspectives.** 3-D view of the fault geometry color-coded with slip and the distribution of aftershocks (black dots, *Ding et al.*, 2023). All segments are labeled. The black arrow indicates the north direction. Red stars represent hypocenters determined by the U.S. Geological Survey (USGS), GEOFOrschungsNetz (GEOFON), the Incorporated Research Institutions for Seismology (IRIS) and the Turkey Disaster and Emergency Management Authority (AFAD).

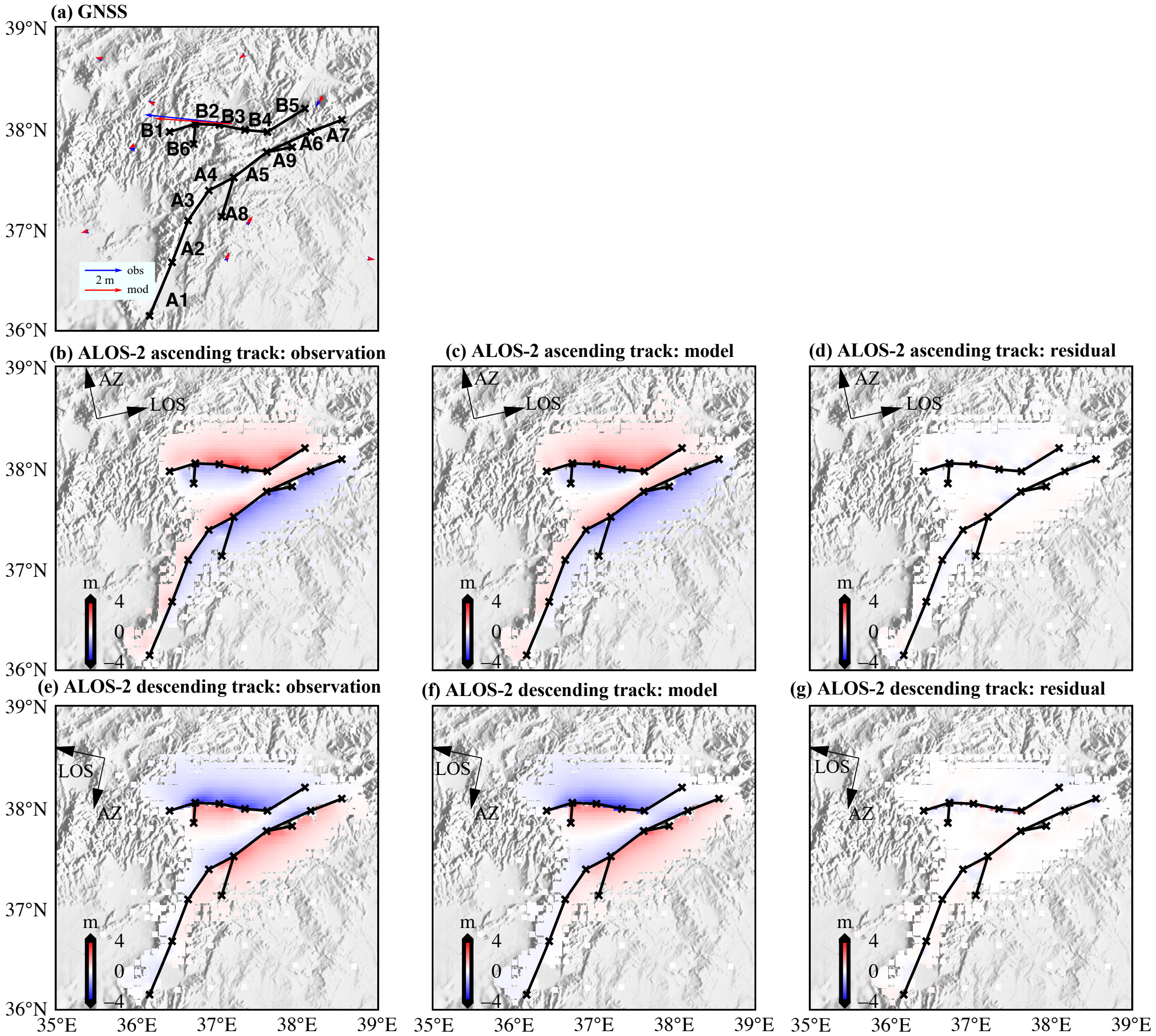

Supplementary Figure 11. The fit to the data for the Bayesian inversion of the fault geometry. (a) The horizontal GNSS observations (blue) versus the corresponding model predictions (red). The ALOS-2 line-of-sight observations (b and e), the corresponding model predictions (c and f), and the unmodeled residuals (d and g) from the ascending and descending tracks are color-coded in the same scale. Fault segments are marked with black solid lines. AZ, azimuthal direction. LOS, line of sight. Positive pixel values indicate motion towards the satellite.

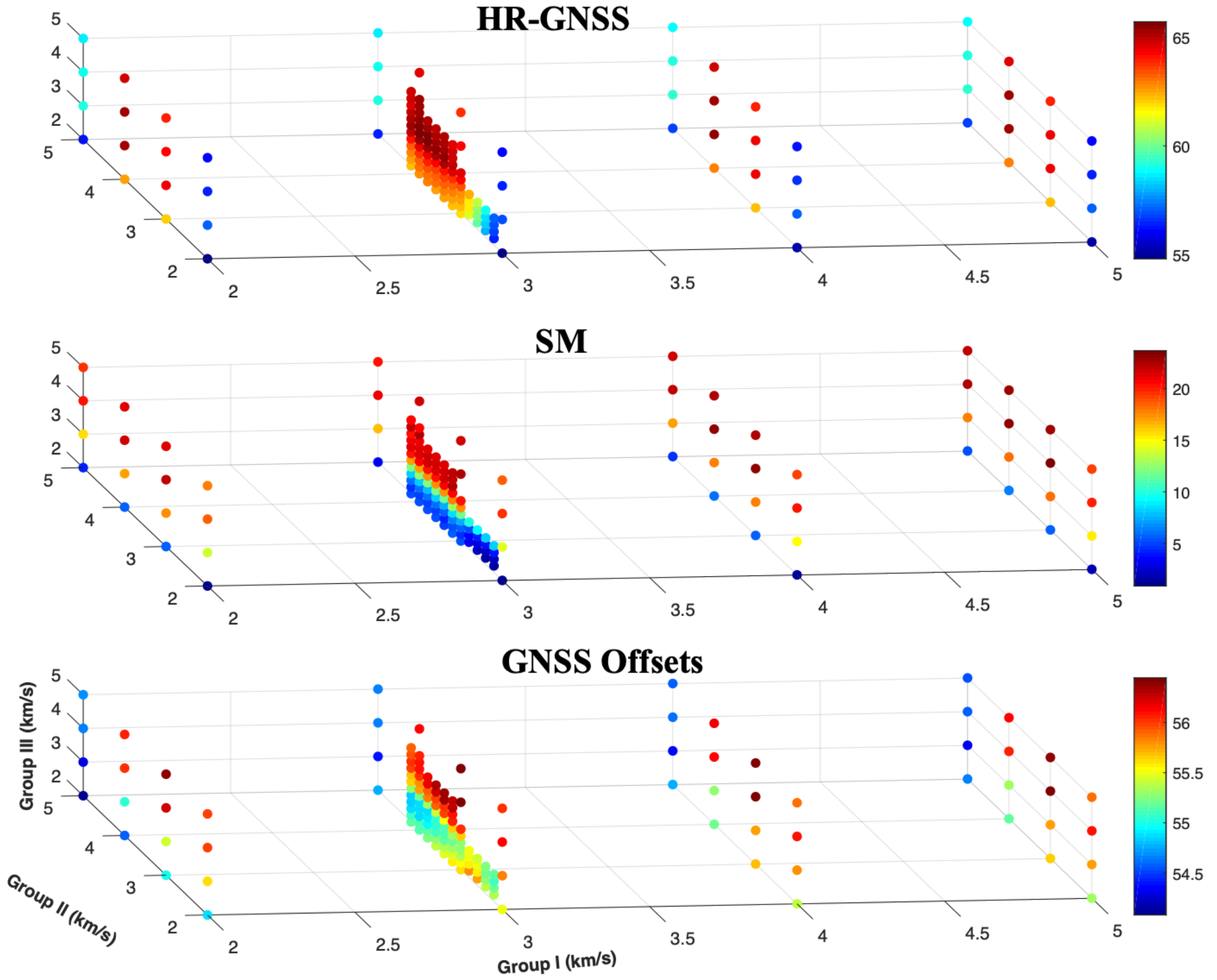

Supplementary Figure 12. **Data fit against rupture speeds of the M7.8 event.** Variance reduction (%) to high-rate GNSS waveforms, strong motion and GNSS offsets as a function of the maximum allowed rupture speed for the M7.8 event.

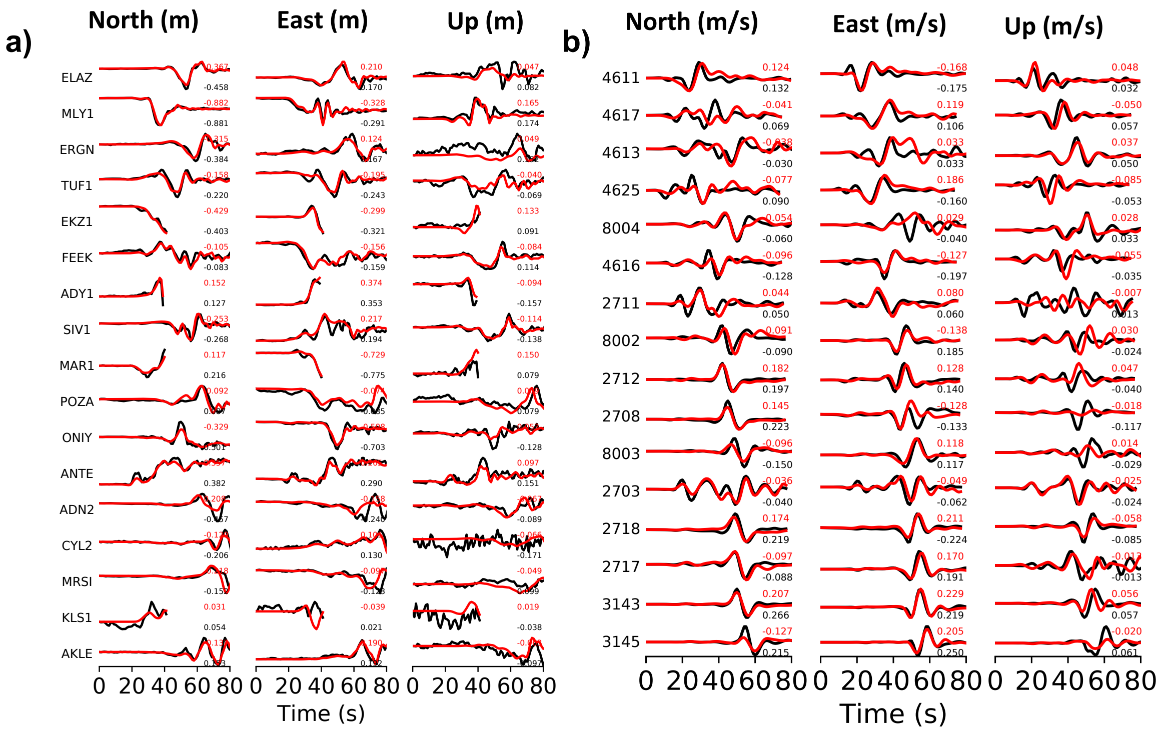

Supplementary Figure 13. **Data fit to the waveform data of the M7.8 event**. GNSS (a) and strong motion (d) waveform observations (black) and fits (red) for the M7.8 event. For each plot, the numbers on the right show the peak amplitude values of the waveform.

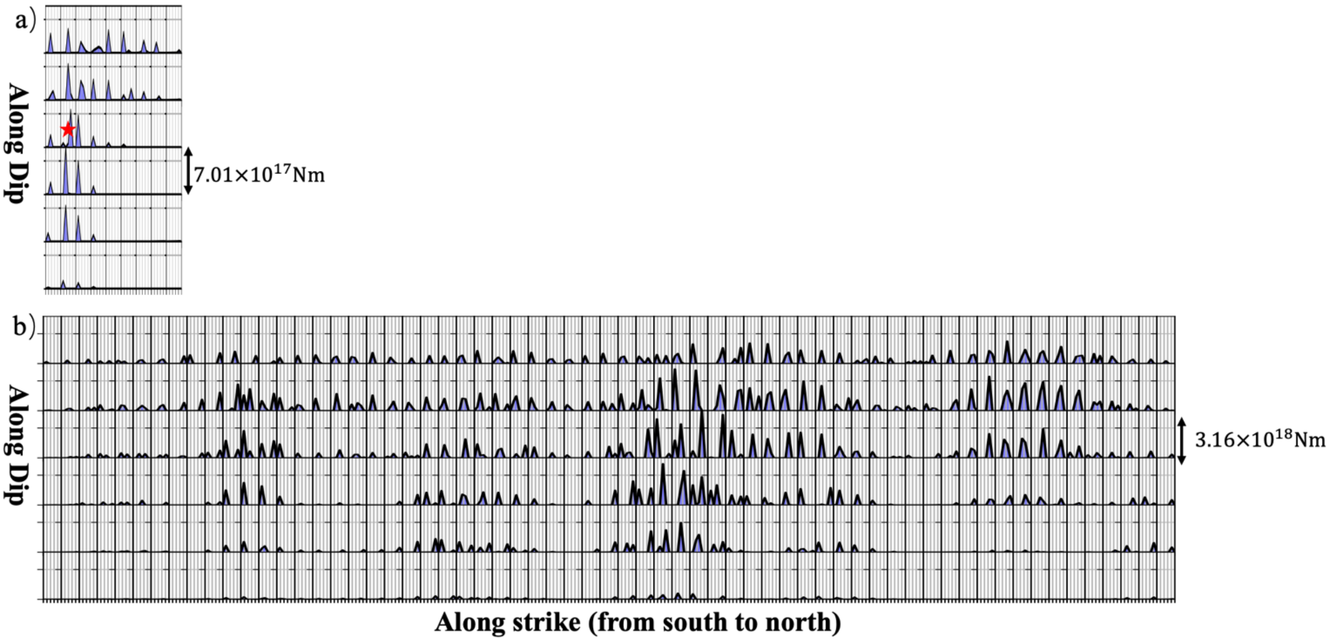

Supplementary Figure 14. **Subfault source time functions for the M7.8 event**. The red star denotes epicencter. a) represents the splay fault NPF and b) shows the main EAF strand.

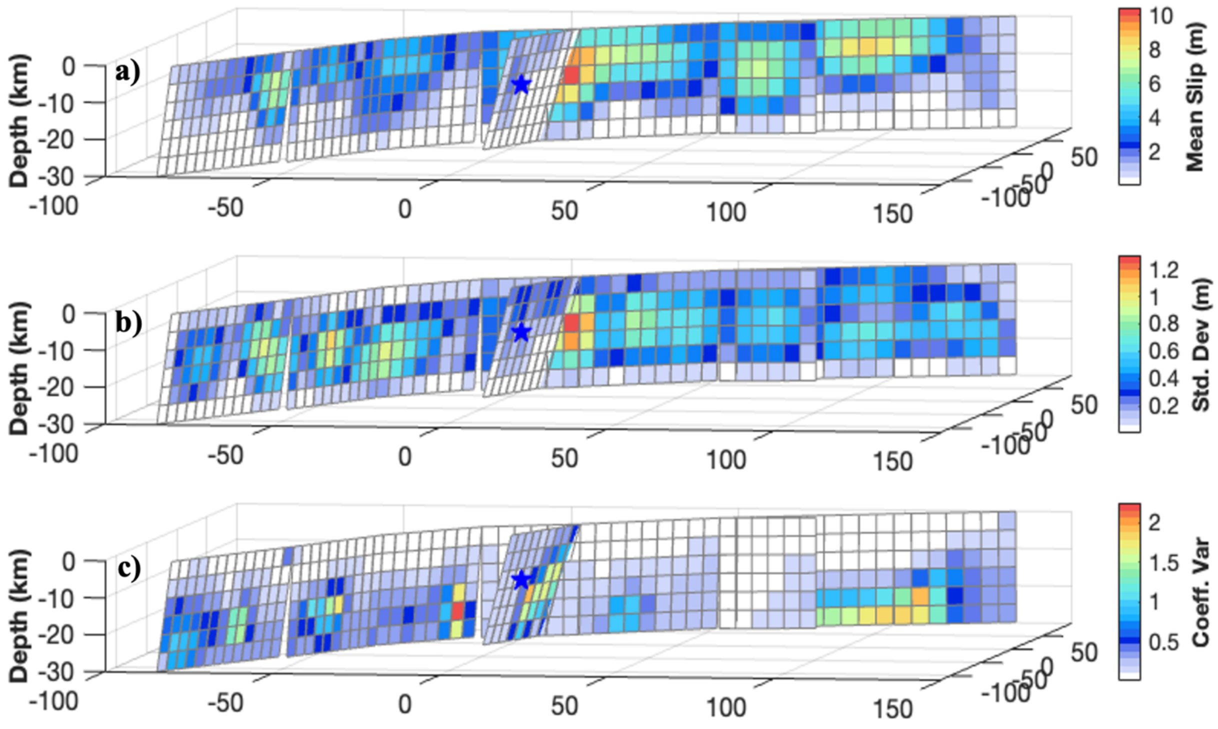

Supplementary Figure 15. Jackknife testing results of removing 20% of the datasets over 100 times for the M7.8 event. a) is the mean slip, b) shows slip standard deviation at 95% confidence interval and c) is coefficient of variation. The blue star denotes epicenter.

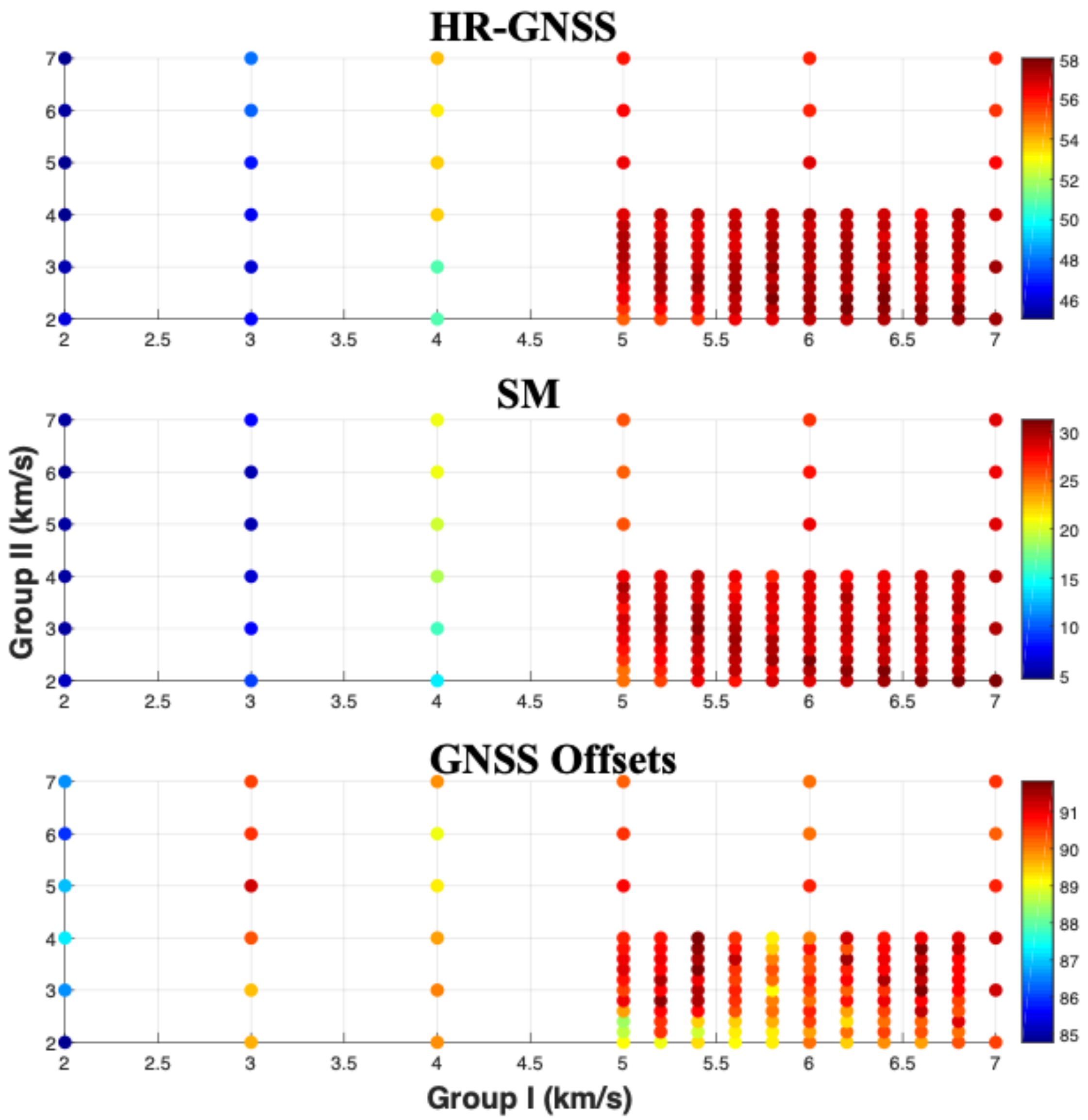

Supplementary Figure 16. **Data fit against rupture speeds of the M7.5 event.** Variance reduction (%) to high-rate GNSS waveforms, strong motion, GNSS offsets as a function of maximum allowed rupture speed for the 2023 M7.5 event.

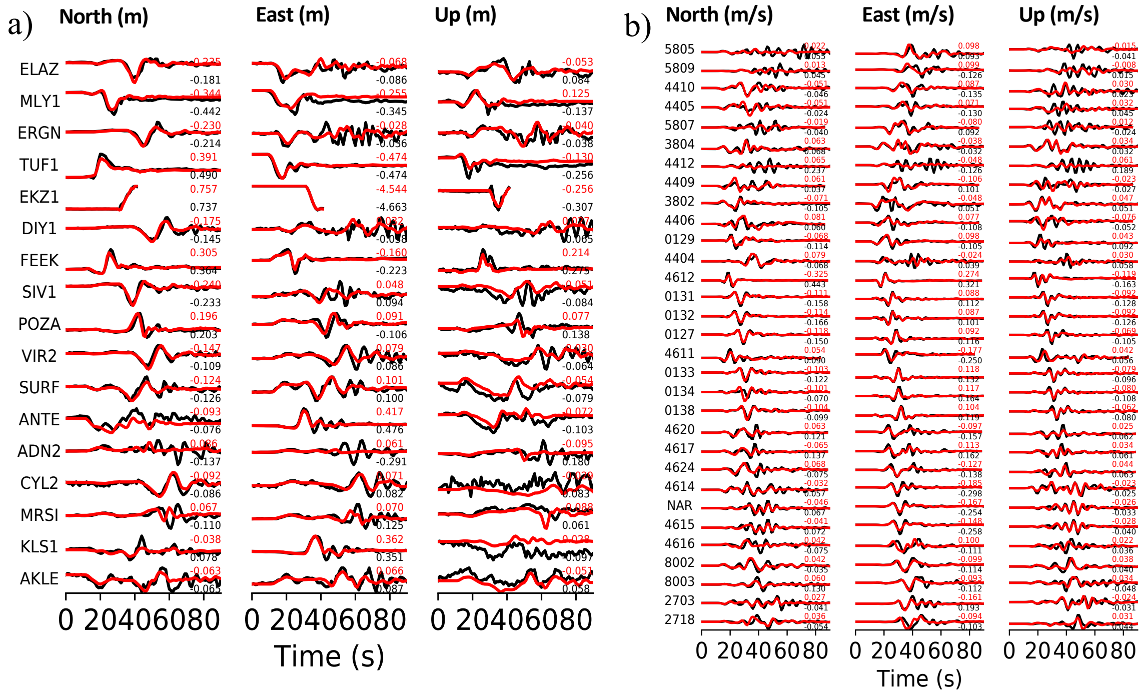

Supplementary Figure 17. **Data fit to the waveform data of the M7.5 event**. GNSS (a) and strong motion (b) waveform observations (black) and fits (red) for the M7.5 event. For each plot, the numbers on the right show the peak amplitude values of the waveform.

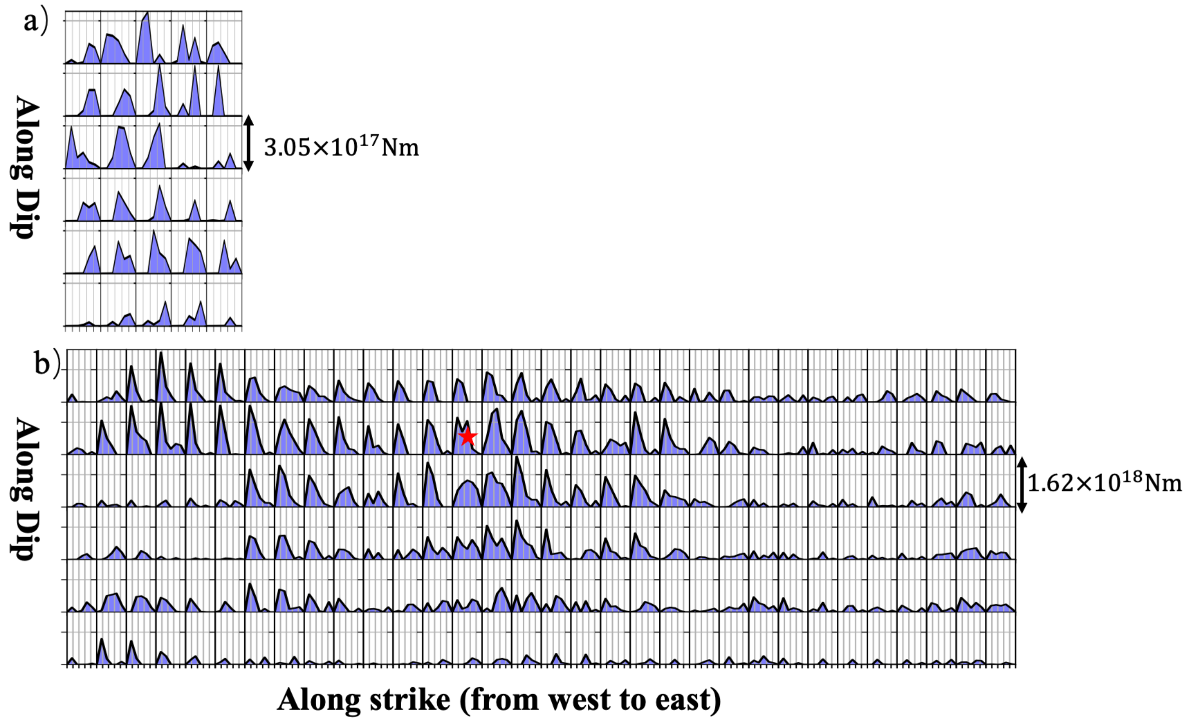

Supplementary Figure 18. **Subfault source time functions for the M7.5 event**. The red star denotes epicencter. a) represents the N-S striking B6 fault and b) shows B1 to B5 main strand.

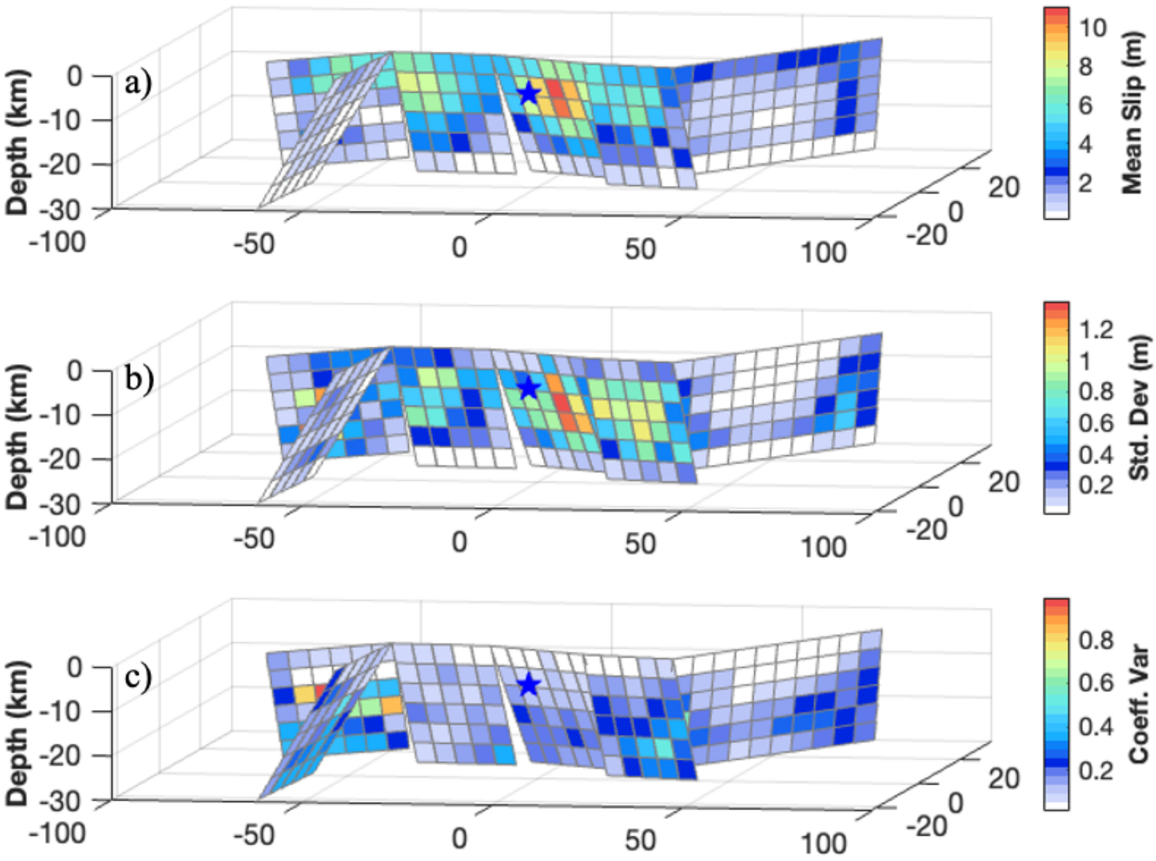

Supplementary Figure 19. **Jackknife testing results of removing 20% of the datasets over 100 times for the M7.5 event**. a) is the mean slip, b) shows slip standard deviation at 95% confidence interval and c) is coefficient of variation. Blue star denotes epicenter.

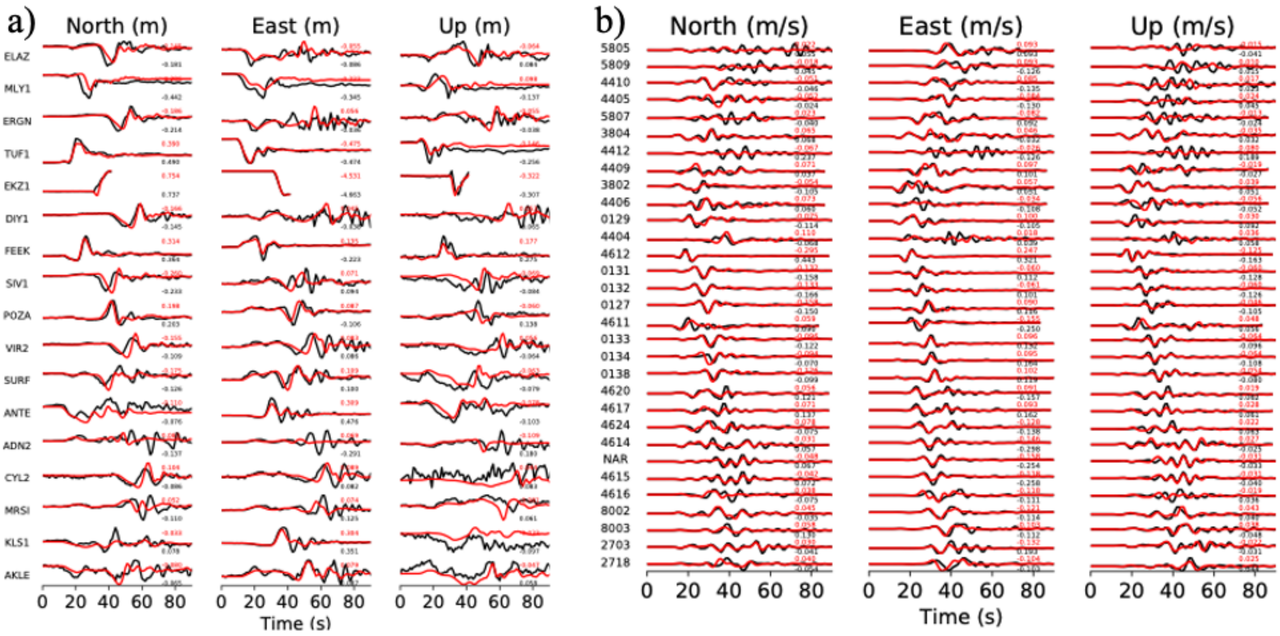

Supplementary Figure 20. **Data fit to the waveform data of the M7.5 event at assumed bilateral rupture speeds**. GNSS (a) and strong motion (b) waveform observations (black) and fits (red) for the M7.5 event assusming only supershear (5.4 km/s) to the westward of the epicenter and 2.8 km/s to the eastward of the epicener.

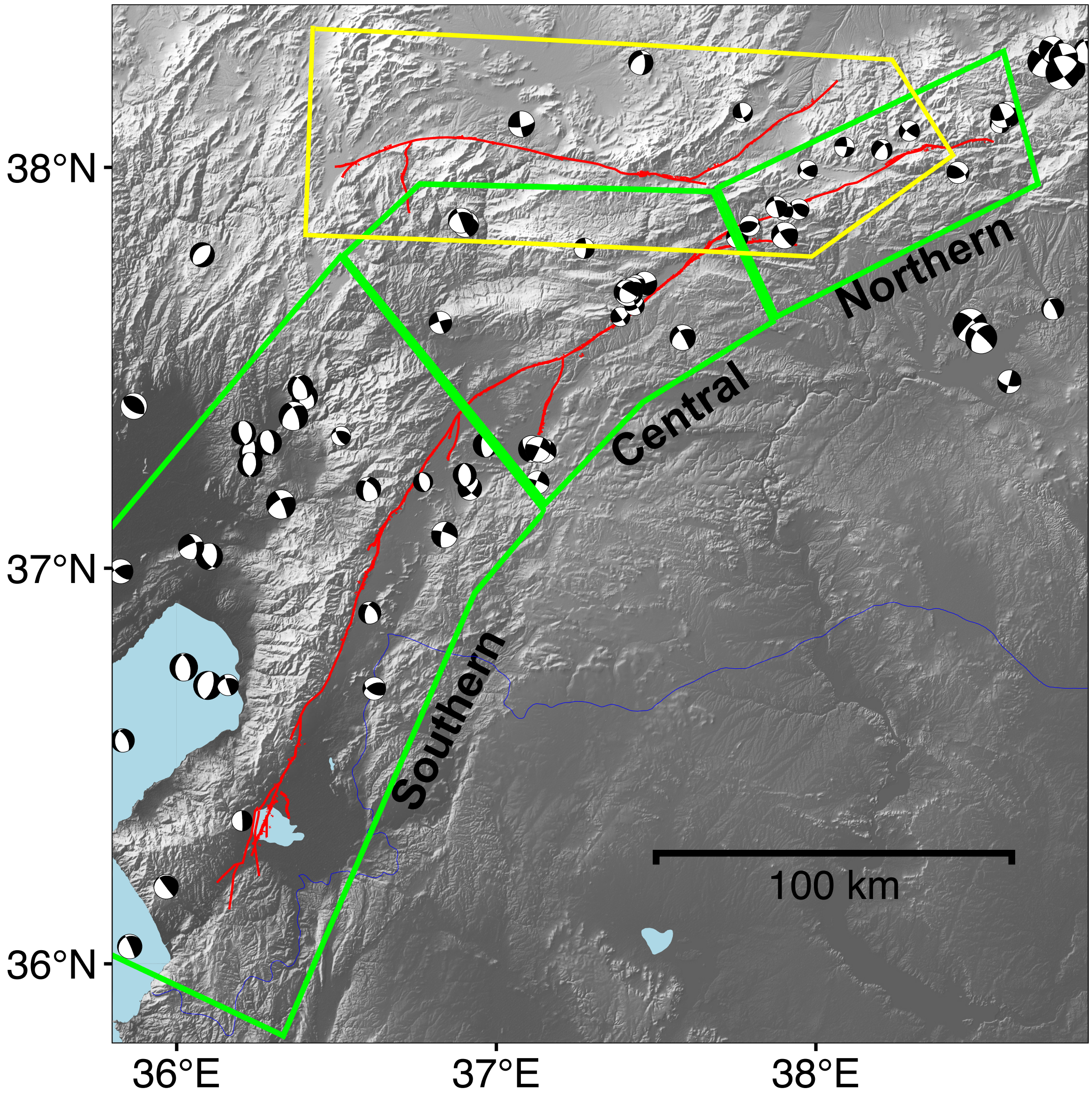

Supplementary Figure 21. Four stress zones. Map illustrating the location and extent of the four stress zones (green and yellow lines), with the coseismic rupture traces mapped (red lines, from *Reitman et al.* [15]) and focal mechanisms derived from *Güvercin et al.* [4].

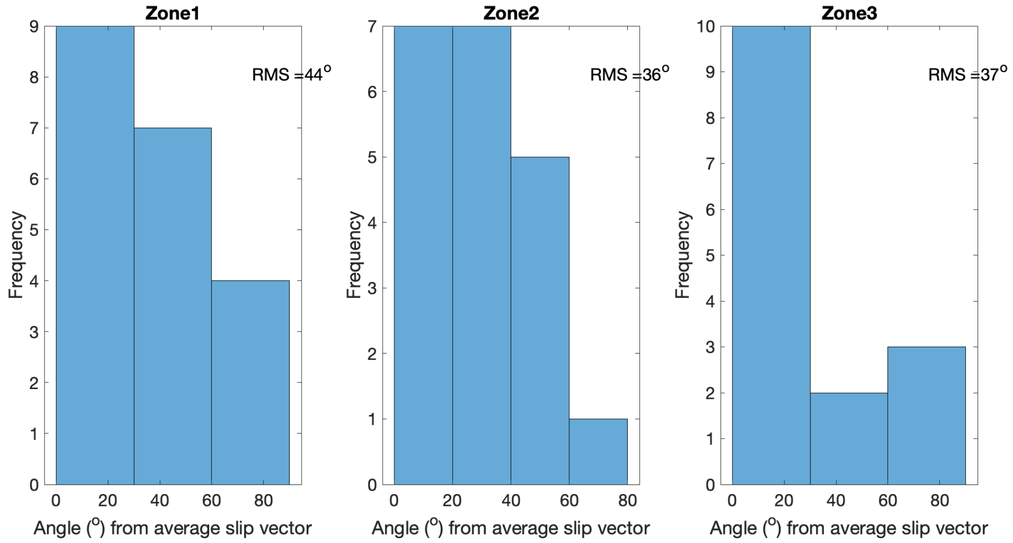

Supplementary Figure 22. Histogram of the root mean squares (RMS) slip vector from the average orientation. Blue is the southern zone, orange is the central zone and yellow is the northern zone. This shows that each stress zone meets and exceeds the minimum requirement of having a 30° RMS difference from the mean mechanism orientation [6] . This demonstrates that each zone has a sufficient diversity in mechanism orientation that is required to reliably estimate the stress tensors.

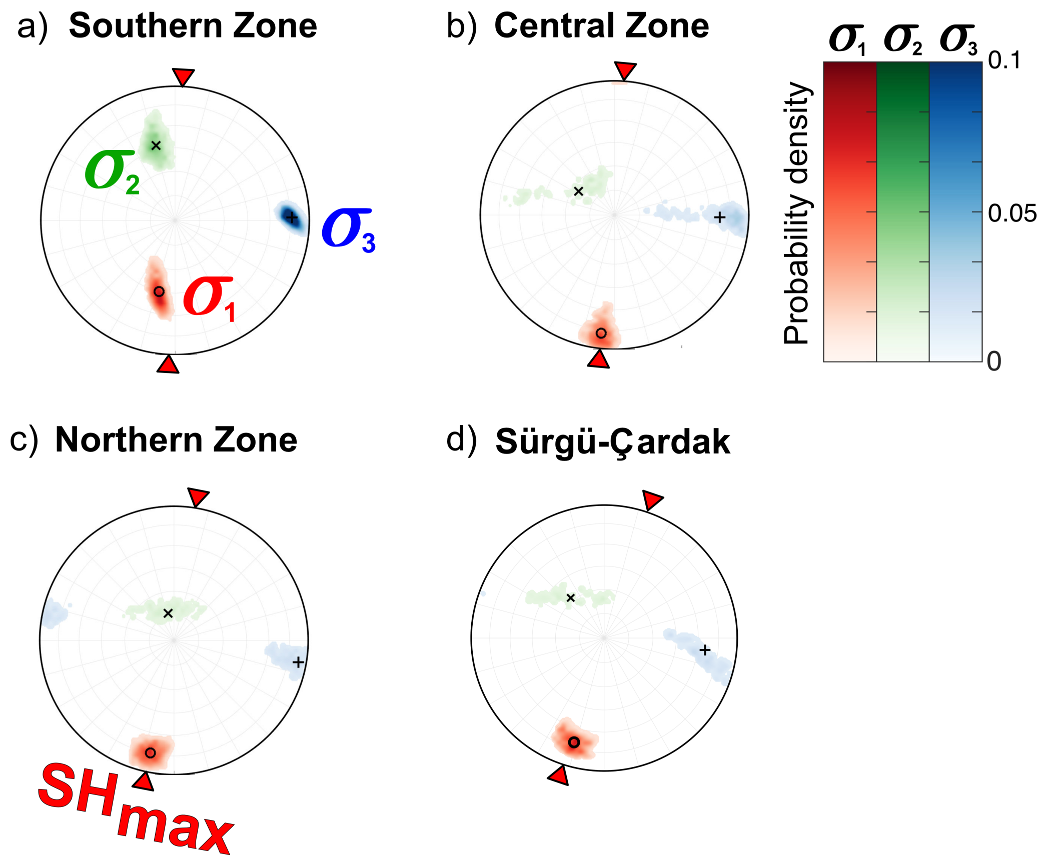

Supplementary Figure 23. Illustration of the principal stresses and their uncertainties for the four zones (a, b, c, d) plotted in lower-hemisphere stereonets from 4000 bootstrap simulations. The colored regions show the probability density; we do not include bins that contain less than 20 model solutions from the bootstrap in order to show regions of stable solutions. Red triangle shows the direction of SHmax [16].

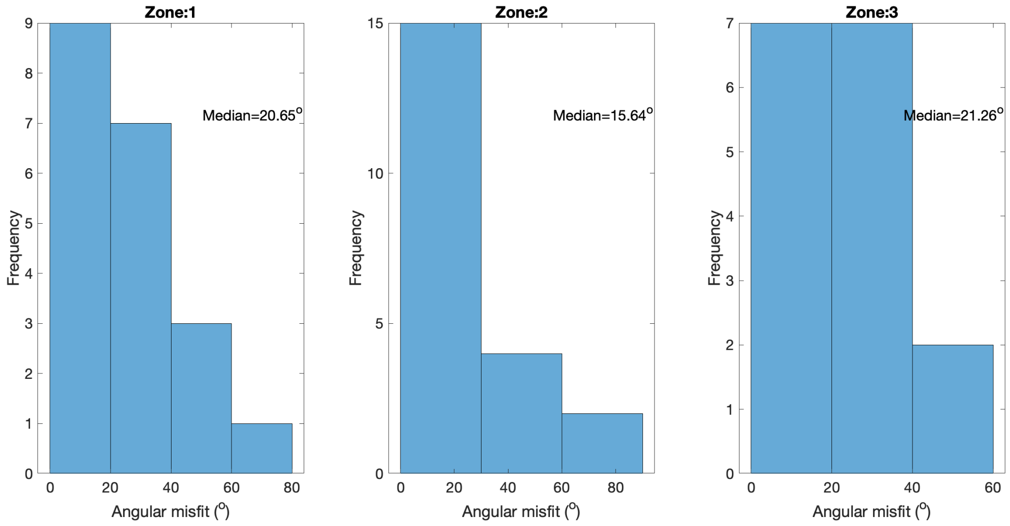

Supplementary Figure 24. Distributions showing the angular misfit between the predicted and observed focal mechanism from our stress tensor inversion for the three zones.

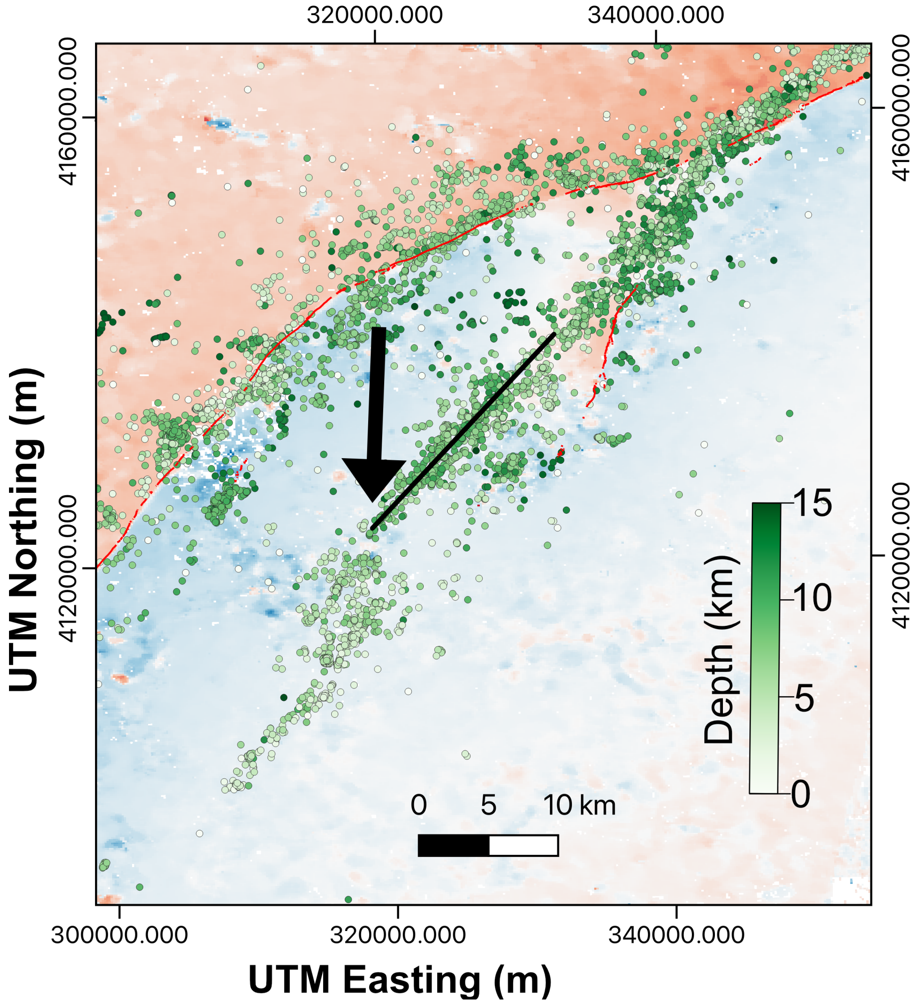

Supplementary Figure 25. **NPF-EAF region with background information**. The aftershock locations color-coded with depth are provided by *Ding et al.* [14], The black line and arrow show the fault strike and *SHmax* (N2.8°E), respectively. Red lines denote the mapped surface rupture traces. Red-blue background colors are the north-south surface displacements. Fault traces are from *Reitman et al.* [15].

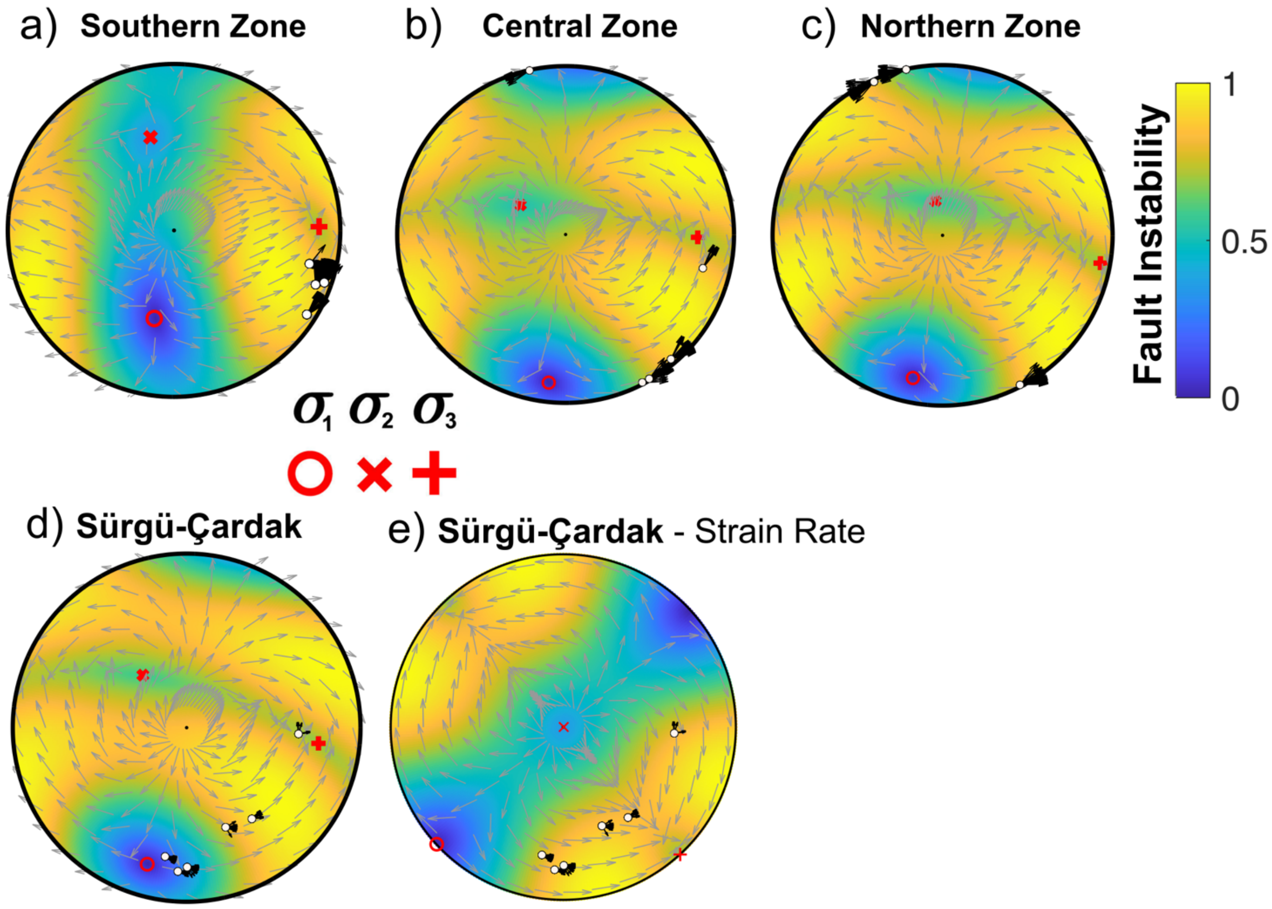

Supplementary Figure 26. **Enlarged pre-stress tensors and fault-slip data for different zones**. Colors show the fault instability.

Supplementary References

1. Leprince, S., Ayoub, F., Klinger, Y. & Avouac, J.-P. Co-Registration of Optically Sensed Images and Correlation (COSI-Corr): an operational methodology for ground deformation measurements. in *2007 IEEE International Geoscience and Remote Sensing Symposium* 1943–1946 (IEEE, 2007). doi:10.1109/IGARSS.2007.4423207
2. Aati, S., Milliner, C. & Avouac, J.-P. A new approach for 2-D and 3-D precise measurements of ground deformation from optimized registration and correlation of optical images and ICA-based filtering of image geometry artifacts. *Remote Sens. Environ.* **277**, 113038 (2022).
3. Rosen, P. A., Gurrola, E., Sacco, G. F. & Zebker, H. The InSAR scientific computing environment. in *Proceedings of the European Conference on Synthetic Aperture Radar, EUSAR* 730–733 (2012).
4. Güvercin, S. E., Karabulut, H., Konca, A. Ö., Doğan, U. & Ergintav, S. Active seismotectonics of the East Anatolian Fault. *Geophys. J. Int.* **230**, 50–69 (2022).
5. Michael, A. J. Determination of stress from slip data: Faults and folds. *J. Geophys. Res. Earth* **89**, 11517–11526 (1984).
6. Hardebeck, J. L. & Hauksson, E. Crustal stress field in Southern California and its implications for fault mechanics. *J. Geophys. Res.* **106**, 859–882 (2001).
7. Vavryčuk, V. Iterative joint inversion for stress and fault orientations from focal mechanisms. *Geophys. J. Int.* **199**, 69–77 (2014).
8. Aster, R. C., Borchers, B. & Thurber, C. H. *Parameter estimation and inverse problems*. (Academic Press, 2011).
9. Hardebeck, J. L. & Michael, A. J. Damped regional-scale stress inversions: Methodology and examples for southern California and the Coalinga aftershock sequence. *J. Geophys. Res. Solid Earth* **111**, (2006).
10. Blewitt, G., Hammond, W. & Kreemer, C. Harnessing the GPS Data Explosion for Interdisciplinary Science. *Eos (Washington. DC).* **99**, (2018).
11. Karabulut, H., Güvercin, S. E., Hollingsworth, J. & Konca, A. Ö. Long silence on the East Anatolian Fault Zone (Southern Turkey) ends with devastating double earthquakes (6 February 2023) over a seismic gap: implications for the seismic potential in the Eastern Mediterranean region. *J. Geol. Soc. London.* **180**, (2023).
12. Ma, Z. *et al.* Space Geodetic Insights to the Dramatic Stress Rotation Induced by the February 2023 Turkey-Syria Earthquake Doublet. *Geophys. Res. Lett.* **51**, (2024).
13. Ou, Q., Lazecky, M., Watson, C. S., Maghsoudi, Y. & Wright, T. 3D displacements and strain from the 2023 February Turkey Earthquakes, version 1. *NERC EDS Cent. Environ. Data Anal.* (2023). doi:0.5285/df93e92a3adc46b9a5c4bd3a547cd242
14. Ding, H. *et al.* High-resolution seismicity imaging and early aftershock migration of the 2023 Kahramanmaraş (SE Türkiye) MW7.9 & 7.8 earthquake doublet. *Earthq. Sci.* **36**, 417–432 (2023).
15. Reitman, N. G. *et al.* Fault rupture mapping of the 6 February 2023 Kahramanmaraş, Türkiye, earthquake sequence from satellite data (ver. 1.1, February 2024). *U.S. Geol. Surv. data release* (2023). doi:https://doi.org/10.5066/P985I7U2
16. Lund, B. & Townend, J. Calculating horizontal stress orientations with full or partial knowledge of the tectonic stress tensor. *Geophys. J. Int.* **170**, 1328–1335 (2007).
