# Supplementary material for: Super-shear ruptures steered by pre-stress heterogeneities during the 2023 Kahramanmaraş earthquake doublet
Source: Nat Commun. 2024 Aug 14;15:7004. doi: 10.1038/s41467-024-51446-y (PMC11325041; doi:10.1038/s41467-024-51446-y)
Supplement: Supplementary file 3 — Description of Additional Supplementary Files [file 41467_2024_51446_MOESM3_ESM.pdf]

Description of Additional Supplementary Files

File Name: Supplementary Movie 1
Description: Rupture propagation of the M7.8 event

File Name: Supplementary Movie 2
Description: Rupture propagation of the M7.5 event
